# Subsite- and histology-specific epidemiology of head and neck cancers in Japan from 2016 to 2019: an application of a new classification of rare cancers

**DOI:** 10.1007/s10147-025-02952-6

**Published:** 2026-04-10

**Authors:** Ryoko Rikitake, Yu Mizushima, Yoko Yamamoto, Takahiro Higashi, Toshihiko Sakai, Akira Kawai, Takahiro Asakage

**Affiliations:** 1 Department of Public Health and Health Policy, Graduate School of Medicine, The University of Tokyo, Tokyo, Japan; 2 Department of Head and Neck Surgery, National Cancer Center Hospital, Tokyo, Japan; 3 Rare Cancer Center, National Cancer Center Hospital, Tokyo, Japan; 4 Department of the Head and Neck Surgery, Institute of Science Tokyo (Formerly Tokyo Medical and Dental University), 1-5-45 Yushima, Bunkyo-Ku, Tokyo, 113-8519 Japan

**Keywords:** Cancer statistics, Head and neck cancer, National cancer registry, Rare cancer, Oral cavity cancer

## Abstract

**Background:**

Head and neck cancers comprise multiple anatomical sites. Due to their rarity, previous studies have often aggregated anatomically or histologically distinct cancers or excluded rare entities altogether. Moreover, the list of head and neck cancer entities has evolved along with the adoption of new pathological insights.

**Methods:**

In our previous study, we proposed a new classification of rare cancers, based on the International Classification of Diseases for Oncology 3.2 coding system, using data from cancer patients at all sites recorded in the National Cancer Registry of Japan. In this study, we created a comprehensive list of head and neck cancers to improve the accuracy of epidemiological reporting using population-based data from 2016 to 2019. Lymphoid diseases were excluded, and only epithelial tumors and sarcomas were included in the analysis.

**Results:**

In Japan, head and neck cancer accounts for approximately 30,000 cases annually, accounting for 2.9% of all cancer cases. The incidence of squamous cell carcinoma arising from the oral cavity and lip was 7.5 cases per 100,000 per year, which does not meet the criterion for a rare cancer. Other head and neck cancers were rare. The most frequent sites were the oral cavity and lip, followed by the larynx, hypopharynx, and oropharynx. The annual number of cases increased during the study period.

**Conclusion:**

Most head and neck cancers were rare during the study period. This result highlights the importance of continued efforts to track incidence trends in parallel with evolving pathological definitions.

**Supplementary Information:**

The online version contains supplementary material available at 10.1007/s10147-025-02952-6.

## Introduction

Head and neck cancers (HNCs) comprise a heterogeneous group of malignancies and collectively rank as the seventh most common cancer worldwide [1]. These cancers typically include malignancies of the oral cavity and lip, pharynx (nasopharynx, oropharynx, and hypopharynx), larynx, major salivary glands, nasal and sinuses, and middle ear, excluding thyroid cancer. Although squamous cell carcinoma of the oral cavity is relatively common, most HNCs are classified as rare due to their low incidence rates when assessed at individual anatomical sites with specific histologies. The anatomical and pathological diversity of HNCs has historically impeded unified epidemiological reporting, particularly the generation of detailed, subsite-specific data. Previous studies have often aggregated anatomically or histologically distinct cancers or excluded rare entities altogether, leading to inconsistent definitions and incomplete epidemiological interpretation [2].

A rare cancer is typically defined as one with an incidence of fewer than six cases per 100,000 persons per year, according to the project Surveillance of Rare Cancers in Europe (RARECARE) [3, 4]. However, the classification of HNC entities continues to evolve alongside new pathological insights, and definitions of rarity may shift with changes in coding systems and diagnostic frameworks.

The implementation of the Japanese National Cancer Registry (NCR) in 2016 established a comprehensive platform for population-based cancer surveillance [5]. This development has created the opportunity to apply a standardized classification system to ensure the systematic capture and interpretation of rare cancers, including HNCs. In our previous study, we proposed a New Classification of Rare Cancers (NCRC) based on the latest International Classification of Diseases for Oncology, third edition, revision 3.2 (ICD-O3.2), and the fifth edition of the World Health Organization (WHO) Classification of Tumors, using NCR data from all cancer sites in Japan [6]. The NCRC enables the standardized classification of HNCs by anatomical site and histology, providing a diagnostic and epidemiological framework applicable in clinical and research settings.

This study aimed to report the incidence and distribution of HNCs in Japan from 2016 to 2019 by leveraging the NCRC framework in conjunction with NCR data. Specifically, we analyzed cancer incidence by anatomical site and histologic subtype, as defined by the NCRC, and described the annual trends in HNC incidence during the study period.

## Patients and methods

### Data sources and study population

We used data from the Japanese NCR to calculate the annual incidence of HNCs. The NCR, launched in 2016, includes all newly diagnosed cancer cases at any primary site in Japan. We identified cases of HNCs diagnosed between 2016 and 2019 involving the following anatomical regions: oral cavity and lip, nasopharynx, oropharynx, hypopharynx, larynx, major salivary glands, nasal cavity and sinuses, and middle ear. Topography codes were assigned using the ICD-O3.2, specifically codes C00x-C14x, C30x-C32x, and C44.2, combined with morphological codes 8000–9582. Malignant tumors (/3) were included. Patients with lymphoid diseases were excluded from the total case count. The HNC histologic subtypes were categorized according to the NCRC using Tier 1 and Tier 2 designations. Patient age and sex were also extracted from the NCR database. The 2015 Japanese standard population data were obtained from the Ministry of Health, Labour, and Welfare, Japan.

### Development of a new classification of rare cancers (NCRC)

The NCRC was developed with two primary objectives: first, to maintain a three-layer hierarchical structure, consisting of Tier 1 (anatomical site), Tier 2 (broad histologic groupings within each site), and Tier 3 (detailed histologic subtypes); and second, to incorporate terminology consistent with the latest WHO Classification of Tumors. Tier 2 and 3 categories were defined by ICD‐O3.2 morphology codes.

A detailed description of the NCRC framework has been previously published [6].

### Data analyses

Crude incidence rates for each HNC type were calculated by dividing the number of new cases by the total population in Japan for each calendar year. Age-adjusted incidence rates were calculated by direct standardization to the 2015 Japan standard population, applying weighted proportions of the corresponding age groups. In the NCRC classification system, rare cancers are those with a crude incidence rate of < 6 cases per 100,000 people per year at the Tier 1 and/or Tier 2 level.

All database processing and statistical analyses were conducted using Stata/MP version 18.1 (StataCorp LLC, College Station, TX, USA).

### Ethical considerations

This study was conducted in accordance with the Cancer Registry Act. The protocol was reviewed and approved by the Data Utilization Committee of the National Cancer Registry Office. The authors are solely responsible for the analysis and interpretation of the NCR data. In accordance with Japanese research ethics guidelines, this study was exempt from institutional review board (IRB) review, as the data handling procedures complied with legal and regulatory requirements. To protect patient confidentiality, counts fewer than 10 (excluding zero) were suppressed and shown as “ < 10” in the table shown in this study.

## Results

A total of 119,982 cases of HNC were registered in Japan from 2016 to 2019, corresponding to an annual average of 29,995.5 cases, which represented 2.9% of all cancers (N = 4,096,363). The NCRC system for classifying HNCs is summarized in Supplementary Table 1. Tier1 includes eight anatomical groupings, while Tier 2 comprises 107 histologic groupings, with allowance for overlapping histologies across multiple primary sites, including site-agnostic Tier 2 histologic categories.

HNCs occurred more frequently in males (N = 87,862, 73.2%) than in females (26.8%). The most frequent age groups were the 65–74 years, accounting for 33.0% of all cases (Table 1). Based on the anatomical subsite, the most common cancers were those of the oral cavity and lip (36.1%), followed by the larynx (17.5%), hypopharynx (16.5%), oropharynx (14.3%), nasal cavity and sinuses (6.6%), major salivary glands (6.2%), nasopharynx (2.6%), and middle ear (0.1%) (Table 1). The age-adjusted annual incidence rate of all HNCs was 23.5 per 100,000 population, standardized to the 2015 Japan population model. Demographic characteristics for each HNC subsite, including the distribution of anatomical subsites for each primary site, are shown in Supplementary Table 2a–c.

**Table 1:** Demographic characteristics of patients with head and neck cancer recorded in the National Cancer Registry between 2016 and 2019

Characteristic		N (%)	Age-adjusted morbidity
Sex			
Male		87,862 (73.2%)	
Female		32,116 (26.8%)	
Sites			
Nasal cavity and sinuses		7918 (6.6%)	
Oral cavity and lip		43,306 (36.1%)	
Nasopharynx		3121 (2.6%)	
Oropharynx		17,170 (14.3%)	
Hypopharynx		19,797 (16.5%)	
Larynx		21,030 (17.5%)	
Major salivary glands		7493 (6.2%)	
Middle ear		147 (0.1%)	
Age			
0–4		19 (0%)	< 0.1
5–9		17 (0%)	< 0.1
10–14		60 (0.1%)	< 0.1
15–19		114 (0.1%)	< 0.1
20–24		287 (0.2%)	< 0.1
25–29		479 (0.4%)	0.1
30–34		830 (0.7%)	0.2
35–39		1277 (1.1%)	0.2
40–44		2483 (2.1%)	0.4
45–49		3945 (3.3%)	0.7
50–54		5558 (4.6%)	1.1
55–59		8381 (7.0%)	1.9
60–64		12,382 (10.3%)	2.9
65–69		19,780 (16.5%)	3.8
70–74		19,834 (16.5%)	3.9
75–79		17,783 (14.8%)	3.3
80–84		13,397 (11.2%)	2.4
85–90		8606 (7.2%)	1.6
90–95		3665 (3.1%)	0.7
95–		1083 (0.8%)	0.2
Total		119,982 (100%)	23.5

^*^Lymphoid disease were omitted from this table

^**^Cases with unknown sex and age were excluded from this table

Supplementary Table 3 presents the crude annual incidence rates for each HNC type based on the NCRC classification. Among Tier 1 anatomical subsites, rates ranged from 0.03 to 8.72 cases per 100,000 population per year. The incidence of squamous cell carcinoma arising from the oral cavity and lip was 7.5 per 100,000 per year, exceeding the threshold for rare cancer. All other HNC entities, by contrast, were considered rare based on the Tier 2 classification.

Table 2 shows the tumor subtype distributions by anatomical sites. The names of the respective histological subtypes were based on NCRC Tier2 (Supplementary Table 1). Histologically, 79.8% of all HNCs were squamous cell carcinomas (SCCs). For reference, the number of lymphomas in each anatomical site and the total number of cases, including lymphoid diseases, are also presented in Table 2. Lymphoid diseases were more frequently observed in the oropharynx, nasopharynx, nasal cavity and sinuses (each approximately 20%).

**Table 2:** Number of cases by tumor histology and primary site

	Nasal cavity and sinuses N (%)	Oral cavity and lip N (%)	Nasopharynx N (%)	Oropharynx N (%)	Hypopharynx N (%)	Larynx N (%)	Major salivary glands N (%)	Total N (%)
Squamous cell carcinoma	4057 (51.2%)	38,200 (88.2%)	0 (0%)	15,353 (89.4%)	18,118 (91.5%)	19,260 (91.6%)	647 (8.6%)	95,725 (79.8%)
Malignant tumor, NOS	818 (10.3%)	2937 (6.8%)	355 (11.4%)	1012 (5.9%)	1357 (6.9%)	1382 (6.6%)	748 (10%)	8638 (7.2%)
Salivary gland type carcinoma	574 (7.2%)	1207 (2.8%)	93 (3.0%)	392 (2.3%)	23 (0.1%)	42 (0.2%)	4867 (65.0%)	7201 (6.0%)
Nasopharyngeal carcinoma	0 (0%)	0 (0%)	1,997 (64.0%)	0 (0%)	0 (0%)	0 (0%)	0 (0%)	1997 (1.7%)
Carcinoma, NOS	145 (1.8%)	219 (0.5%)	181 (5.8%)	151 (0.9%)	122 (0.6%)	129 (0.6%)	383 (5.1%)	1331 (1.1%)
Mucosa and extracutaneous melanoma	881 (11.1%)	217 (0.5%)	< 10 (−)	27 (0.2%)	< 10 (−)	0 (0%)	< 10 (−)	1149 (1.0%)
Adenocarcinoma, NOS	0 (0%)	0 (0%)	0 (0%)	0 (0%)	0 (0%)	43 (0.2%)	663 (8.8%)	706 (0.6%)
Adenocarcinoma	271 (3.4%)	180 (0.4%)	47 (1.5%)	84 (0.5%)	32 (0.2%)	0 (0%)	0 (0%)	616 (0.5%)
Sarcoma	284 (3.6%)	131 (0.3%)	10 (0.3%)	26 (0.2%)	36 (0.2%)	68 (0.3%)	28 (0.4%)	586 (0.5%)
Olfactory neuroblastoma	496 (6.3%)	0 (0%)	0 (0%)	0 (0%)	0 (0%)	0 (0%)	0 (0%)	496 (0.4%)
Lymphoepithelioma(-like) carcinoma	0 (0%)	12 (0%)	304 (9.7%)	38 (0.2%)	33 (0.2%)	< 10 (−)	48 (0.6%)	440 (0.4%)
Neuroendocrine carcinoma, NOS	200 (2.5%)	22 (0.1%)	11 (0.4%)	46 (0.3%)	26 (0.1%)	66 (0.3%)	36 (0.5%)	409 (0.3%)
Undifferentiated carcinoma, NOS	136 (1.7%)	33 (0.1%)	93 (3.0%)	27 (0.2%)	28 (0.1%)	< 10 (−)	27 (0.4%)	351 (0.3%)
Odontogenic tumor	0 (0%)	127 (0.3%)	0 (0%)	0 (0%)	0 (0%)	0 (0%)	0 (0%)	127 (0.1%)
Carcinosarcoma / Sarcomatoid carcinoma, NOS	27 (0.3%)	13 (0%)	< 10 (−)	< 10 (−)	12 (0.1%)	14 (0.1%)	28 (0.4%)	103 (0.1%)
Neuroendocrine tumor, NOS	14 (0.2%)	< 10 (−)	< 10 (−)	< 10 (−)	< 10 (−)	13 (0.1%)	< 10 (−)	35 (0%)
Nasopharyngeal papillary adenocarcinoma	0 (0%)	0 (0%)	18 (0.6%)	0 (0%)	0 (0%)	0 (0%)	0 (0%)	18 (0%)
Histiocytic and Dendritic cell neoplasms	< 10 (−)	< 10 (−)	< 10 (−)	< 10 (−)	< 10 (−)	0 (0%)	< 10 (−)	16 (0%)
Middle ear Adenoma	0 (0%)	0 (0%)	0 (0%)	0 (0%)	0 (0%)	0 (0%)	0 (0%)	12 (0%)
Acute myeloid leukaemia and related precursor neoplasms	< 10 (−)	< 10 (−)	0 (0%)	0 (0%)	0 (0%)	0 (0%)	0 (0%)	< 10 (−)
Mixed neuroendocrine non-neuroendocrine neoplasm	0 (0%)	0 (0%)	0 (0%)	< 10 (−)	< 10 (−)	< 10 (−)	0 (0%)	< 10 −)
Germ cell associated tumor	< 10 (−)	0 (0%)	0 (0%)	0 (0%)	0 (0%)	0 (0%)	0 (0%)	< 10 (−)
Teratocarcinosarcoma	< 10 (−)	0 (0%)	0 (0%)	0 (0%)	0 (0%)	0 (0%)	0 (0%)	< 10 (−)
Neuroblastoma	0 (0%)	0 (0%)	0 (0%)	< 10 (−)	< 10 (−)	0 (0%)	0 (0%)	< 10 (−)
Paraganglioma, NOS, malignant	< 10 (−)	0 (0%)	0 (0%)	0 (0%)	0 (0%)	0 (0%)	0 (0%)	< 10 (−)
n/a	0 (0%)	0 (0%)	0 (0%)	0 (0%)	0 (0%)	0 (0%)	< 10 (−)	< 10 (−)
Total	7,918 (100%)	43,306 (100%)	3,121 (100%)	17,170 (100%)	19,797 (100%)	21,030 (100%)	7,493 (100%)	119,982 (100%)
Lymphoid diseases	2,652	823	830	4,372	98	123	1,123	10,025
Total (include Lymphoid disease)	10,570	44,129	3,951	21,542	19,895	21,153	8,616	1,30,007

*Middle ear cancer cases were omitted from this table (due to small number of cases). Therefore, the sum of cases in this table does not match the number in the "Total" column

**Please refer to Supplementary Table 1 (NCRC Tier2) for the names of the respective histological subtypes

***Less than 10 cases (except for 0) of given histology and primary sites are represented as “ < 10.”

**Fig. 1:**
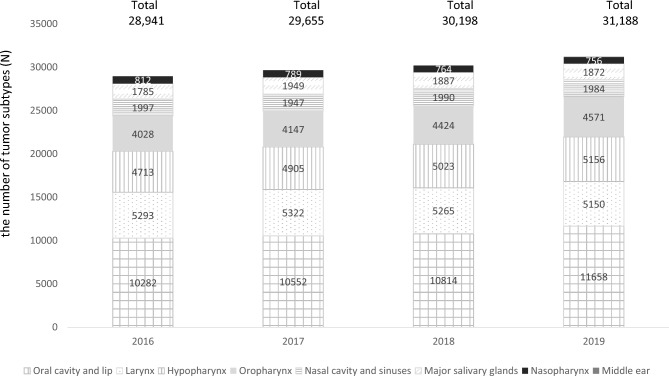
Annual trends in head and neck cancer statistics recorded in the National Cancer Registry between 2016 and 2019

We also examined the annual trends in the number of HNC cases (Fig. 1). The total number of HNCs increased from 28,941 cases in 2016 to 31,188 cases in 2019. The annual trends in cancer case numbers for each HNC primary site are also shown in Fig. 1. The number of oral cavity and lip cancers has increased from 10,282 in 2016 to 11,658 in 2019. Oropharyngeal cancers increased from 4028 to 4571 cases, and hypopharyngeal cancers increased from 4713 to 5156 cases during the same period. The incidence of cancers in the larynx, nasal cavity and sinuses, and nasopharynx remained relatively stable. In addition, Supplementary Table 4 presents the annual changes in histologic subtypes with sufficient case numbers.

## Discussion

This study is the first to use the NCRC in conjunction with data from the NCR to report the incidence of HNCs by site in Japan. The epidemiology of HNCs is complex, and the rarity of its multiple subtypes contributes to underreporting and inconsistent surveillance. Our study adopted a comprehensive, standardized approach for the classification and quantification of HNCs, addressing key challenges in population-based cancer registration.

In Japan, HNCs account for approximately 30,000 cases annually, representing 2.9% of all cancers registered between 2016 and 2019. The age-adjusted annual incidence rate of HNCs was 23.5 per 100,000 population, standardized to the 2015 Japanese population model. As sarcomas, which have often been excluded in previous reports, are among the diseases treated by head and neck surgeons, they were included in this study. Patients with lymphoid diseases were excluded. For comparison, a previous study that included only epithelial malignancies (ICD-O3 morphology codes 8000–8574) reported incidence rates of 12.42 per 100,000 men and 3.71 per 100,000 women in 2015, based on population-based cancer registry data [7].

Only SCC of the oral cavity and lip exceeded the rare-cancer rate of 6 cases per 100,000 per year, with a crude incidence of 7.5 per 100,000. All other HNC types were classified as rare. The most common subsites, in order of frequency, were the oral cavity and lip (36.1%), followed by the larynx (17.5%), hypopharynx (16.5%), oropharynx (14.3%), nasal cavity and sinuses (6.6%), major salivary glands (6.2%), nasopharynx (2.6%), and middle ear (0.1%). Globally, approximately 50% of HNCs arise in the oral cavity [1]. Compared with international or European data, Japan exhibits a relatively higher proportion of hypopharyngeal cancers and a lower proportion of nasopharyngeal cancers within the pharyngeal category [1, 3, 8].

HNC incidence was higher in men, and in older adults, consistent with global epidemiological patterns. From 2016 to 2019, the total number of HNC cases showed a gradual upward trend. Given the aging population of Japan, this increasing trend is expected to continue. The predominance of SCC in our cohort was also consistent with international observations [9]. Of particular concern is the rising incidence of oropharyngeal and oral cavity cancers, both of which demonstrated noticeable increases during the study period. This increase mirrors global trends associated with human papillomavirus (HPV) infection, especially in high-income countries [10]. Conversely, the stable or declining incidence of laryngeal cancer likely reflects reduced exposure to traditional risk factors, such as tobacco and alcohol. This may be related to public health efforts promoting anti-smoking policies and the general decline in smoking prevalence in Japan [11]. Both oral cavity and hypopharyngeal cancers showed increasing trends, which may be partly explained by the expanded use of narrow-band imaging (NBI) during gastrointestinal endoscopy in recent years. This technique has enabled gastroenterologists to detect early-stage lesions in the oral cavity and pharynx during routine examinations, leading to a higher number of detected cases [12, 13]. In East Asian populations, including the Japanese, more than 30% of individuals have an inactive form of aldehyde dehydrogenase 2 (ALDH2), which results in impaired alcohol metabolism and increased carcinogenic risk [14]. Geographic, genetic, and environmental factors also likely contribute to the observed variation in HNC incidence and warrant further investigation [15].

The NCRC framework has enhanced the identification of rare HNC types, such as salivary gland adenocarcinomas and neuroendocrine tumors, underscoring the need for greater precision in cancer registration and policymaking. In Japan, incidence rates by site for HNCs with corresponding histological classifications have not been previously reported at the national level, largely because of the complexity of assigning cases to anatomically and histologically distinct categories. In this study, we clarified the incidence rates by site in the Japanese population for the first time, using all registered cancer cases and providing detailed distributions by sex and age. The NCRC framework emphasizes standardized classification according to the latest pathological criteria (ICD-O3.2), enabling a more accurate enumeration of cancer incidence by both anatomical subsite and histological type.

This study had several limitations. First, the observation period was relatively short (four years), limiting the ability to assess long-term incidence trends. Moreover, data beyond 2019 were not included; during the COVID-19 pandemic period (from 2020 onward), a significant decline in cancer diagnoses, including HNCs, has been reported and is expected [16]. Second, survival data were not available for the years analyzed, precluding prognostic evaluation. Third, although our study included all histologies recorded in the NCR database, we did not analyze detailed clinical variables, such as cancer stage or treatment modality. The NCR includes data on summary stage (e.g., localized, regional, distant), route of detection (e.g., screening, symptomatic presentation), and initial treatment (such as surgery, radiation, chemotherapy) [17], but we did not evaluate these parameters in the current study. Future research should assess the consistency and completeness of these data across hospitals in Japan.

Finally, we could not examine patient-level clinical background, including etiological risk factors or biomarker test results (e.g., HPV testing results). To obtain such information, complementary registries, such as hospital-based cancer registries maintained by designated cancer care hospitals under the Ministry of Health, Labour and Welfare, may be useful [18]. Compared to hospital-based registries, the strength of the NCR lies in its nationwide coverage and population-level completeness, making it a powerful resource for epidemiologic surveillance.

## Conclusion

Most HNCs were classified as rare, according to the criteria defined by the NCRC. Analysis of data from the NCR revealed an increasing trend in the incidence of HNCs in Japan during the current study period. These results highlight the importance of continued surveillance to monitor temporal changes in incidence, particularly in the context of evolving pathological classifications. Adopting the NCRC framework may improve the identification and classification of rare cancers, and support the development of targeted public health strategies. This study contributes new, population-based evidence on the incidence of HNCs by site in Japan and provides a standardized baseline for future epidemiological research on rare cancers.

## Supplementary Information

Below is the link to the electronic supplementary material.

Supplementary file1 (XLSX 36 KB)

## Data Availability

The analysis used individual-level data from the National Cancer Registry in Japan, which are not publicly available due to legal and privacy restrictions. Access may be granted with the approval from the Data Utilization Committee of the National Cancer Registry Office.
